# Heterogeneity affects the differentiation potential of dental follicle stem cells through the TGF-β signaling pathway

**DOI:** 10.1080/21655979.2021.2009974

**Published:** 2021-12-18

**Authors:** Meng Zhaosong, Fu Na, Guo Shuling, Liu Jiacheng, Wei Ran

**Affiliations:** a Hospital of Stomatology, Tianjin Medical University, Tianjin, China; b School of Stomatology, Tianjin Medical University, Tianjin, China

**Keywords:** Dental stem cells, heterogeneity, cell differentiation

## Abstract

Adult mesenchymal stem cells play an important role in maintaining organ homeostasis owing to their unique ability to generate more specialized cell populations in a coordinated manner. Adult mesenchymal stem cells are heterogeneous, a feature that is essential for their functions. However, studies have not elucidated how heterogeneity of mesenchymal stem cells affects their differentiation capacity. The current study thus explored the heterogeneous Dental Follicle Stem Cells (DFSCs). A previous study by our research group reported that selecting sub-clones can cause artificial damage of the heterogeneous microenvironment of DFSCs. The finds showed a decrease in differentiation capacity of the three subclones, although the underlying mechanism was not elucidated. In this study, cells were harvested and prepared for gene expression microarray analysis. Sequence data was used in gene ontology and pathway enrichment analysis. The results showed that downregulation of the TGF-β signaling pathway was the main cause of changes in differentiation of sub-clones. Additional analyses revealed that the Hippo pathway, WNT pathway and signaling pathways regulating the pluripotency of stem cells were also implicated in these changes, through a cross talk with TGF-β signaling pathway through Bmp2, Bmp4, and Bambi. *In vivo* implantation experiments and osteogenic induction showed that differentiation capacity of DFSCs was significantly reduced in the sub-clones. In summary, the findings of the current study show that differentiation potential of DFSCs is correlated with the heterogeneous microenvironment and TGF-β signaling pathway significantly modulates these biological processes.

## GRAPHICAL ABSTRACT

**Figure:**
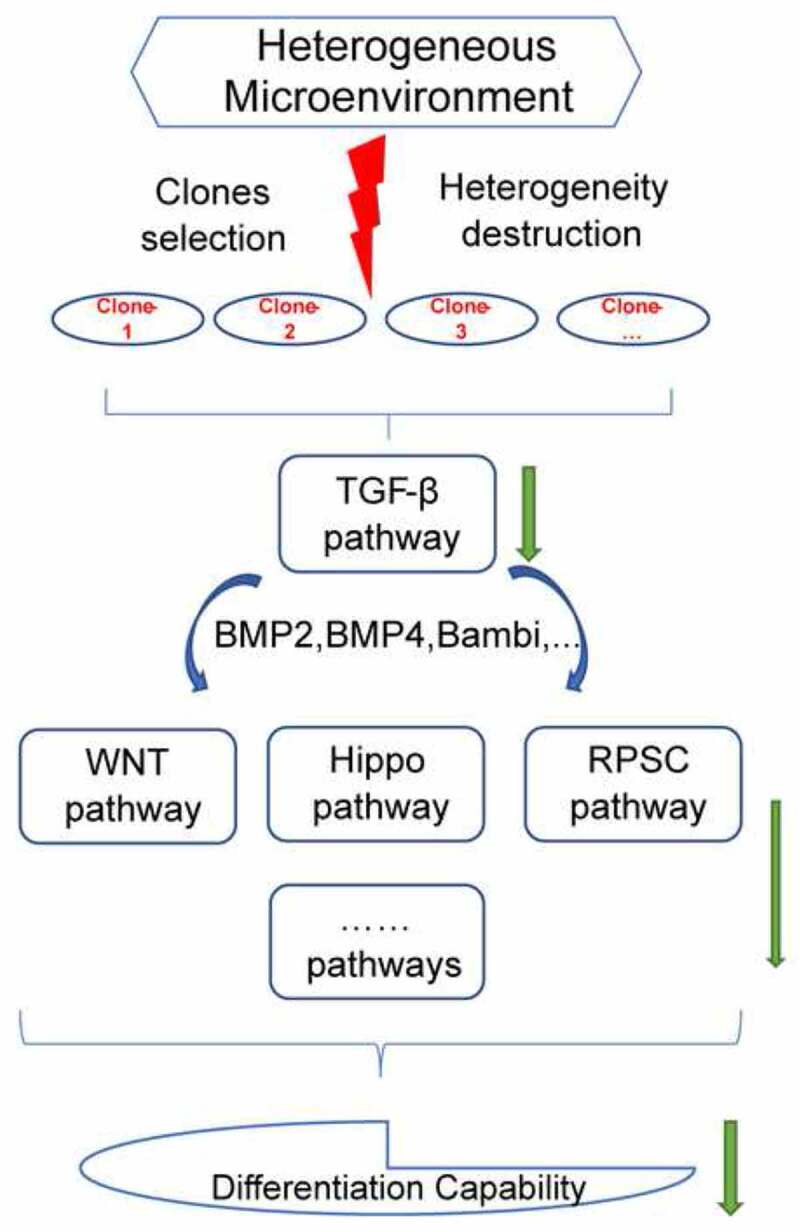

## 1. Introduction

Stem cells play an important role in organ homeostasis, tissue regeneration and disease therapy owing to their multipotency and an infinite capacity of self-renewal [1]. However, the heterogeneous microenvironment of stem cells plays an important in these functions [2,3]. Heterogeneity means cell to cell variation in genomes, transcriptomes, and/or epigenomes, resulting in a variety of subpopulations inner the stem cells [4]. Heterogeneity of stem cells can activate quiescent stem cells or particular subpopulations of stem cells in response to stimulation targeting the body *in vivo* [5]. In addition, heterogeneity allows stem cells to respond to different signals and differentiate into corresponding cells *in vitro* [6,7]. Several studies report that heterogeneity may be important in cell differentiation given that differentiated cells are heterogeneous [8]. However, the mechanism through which heterogeneity affects differentiation of stem cells has not been fully elucidated. Therefore, there is need to explore the underlying mechanism of modulation of differentiation by heterogeneity using appropriate methods.

Dental stem cells are promising seeding cells for tissue engineering and stem cell therapy because of their multipotent differentiation capacity [9]. In addition, several studies have shown that dental stem cells can be used in a wide range of applications including, tooth regeneration [10], bone regeneration [11], nervous disease [12], immune disease [13] and inflammation control [14]. Moreover, dental stem cells are easy to store, and their properties are consistent before and after cryopreservation. DFSCs are isolated from the extraction of wisdom teeth, and are unique types of dental stem cells. This is because DFSCs have high exceptional multipotent differentiation potential (even better embryonic stemness characteristics and osteogenesis ability) and are easy to used but not limited by ethical restrictions [15,16]. Furthermore, DFSCs are heterogeneous as embryonic stem cells and other mesenchymal stem cells [17,18]. However, only a few studies have explored heterogeneity of DFSCs, fewer on the role in differentiation.

In this study, we tried to disrupt heterogeneous microenvironments by selecting sub-clones from DFSCs. The sub-clones were induced *in vitro* and *in vivo* to evaluate the differentiation capability, comparing heterogeneous DFSCs. The gene expression array was used to analyze the possible mechanism of differentiation capability change. Finally, we hope to explore the possible effects of heterogeneity on sub-clones’ differentiation and its preliminary mechanism.

## 2. Materials and methods

### 2.1. Isolation procedure of sub-clones

**Figure 1.**
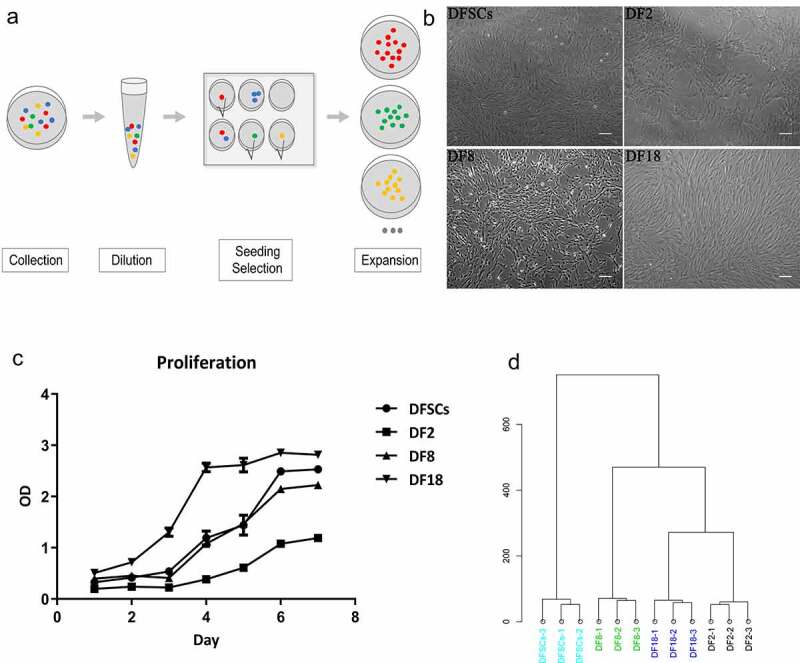
The selection protocol for sub-clones. (a) The selection procedure for single sub-clone. (b) Images of DFSCs and three sub-clones. (Scale bar: 100 μm). (c) Cell proliferation assay. CCK-8 assay were used to evaluate the proliferation capability of 3 sub-clones and DFSCs. Error bar indicated SEM (n = 3). DF18 showed the highest proliferation activity while DF2 stayed at a relatively quiescent level on the contrary; DF8 and DFSCs showed similar proliferation level. (d) Hierarchical clustering analysis. Hierarchical clustering of Gene Expression Array for sub-clones and DFSCs. Sub-clones and DFSCs were proved to be different cluster

DFSCs and three sub-clones were isolated and cultured as previously described [3,18]. Specific selection process is shown in Figure 1(a). The limited dilution method was used to select a single cell and for amplification of the clones. The first passage of rat-DFSCs was then digested, diluted and seeded into a 96-well plate. Wells with only one cell was chosen for subsequent analysis. The cells were then passaged in a 48-well plate, 24-well, 12-well or 6-well plate, in a T-25 (Costar, MA, USA) and T-75 cell culture flask. In addition, sub-clones that showed no expansion were excluded. There sub-clones (DF2, DF8 and DF18) were selected from DFSCs (Figure 1(b)).

### 2.2. Cell Proliferation analysis

Cell Counting Kit-8 (CCK-8, Dojindo, Japan) was utilized to quantitatively evaluate the viability of cells. 2*10^3^ cells were cultivated on 96-well plates (Thermo, USA). The original cultivation medium was replaced by 120 ml α-MEM with 10% FBS containing 12 ml CCK-8 for each well of 96-well plate at the same time of consecutive 7 d. After incubation at 37°C for 4 h, 100 ml of the above solution was taken from each sample and added to one well of another new 96-well plate. Three parallel replicates were prepared and the absorbance at 450 nm was detected using a spectrophotometer (Thermo, USA).

### 2.3. RNA extraction and gene expression array

Total RNA was extracted following a standard protocol and Gene Expression Microarray protocol (Agilent, USA) was used for microarray analysis. *Analysis of differentially expressed genes*: Raw data were normalized using the Quantile algorithm in the Gene Spring Software 11.0 (Agilent Technologies, Santa Clara, CA, US). Gene Ontology (GO) and the Kyoto Encyclopedia of Genes and Genomes (KEGG) enrichment analyses were conducted on the Differentially Expressed Genes (DEGs) using the Cluster Profiler package in R (fold change > 2, p-Adjust Method = ‘BH’, cutoff value for p = 0.01, cutoff value for q = 0.05). Moreover, GO and KEGG enrichment analyses and annotations were performed using the Database for Annotation, Visualization and Integrated Discovery (DAVID) (https://david.ncifcrf.gov/) and the KEGG database (version: 11.04.2021). *Pathway enrichment analysis*: Pathway enrichment network analysis and visualization of *mesenchymal cell differentiation* and *odontogenesis* were performed using the ClueGO and CluePedia plug-ins in Cytoscape 3.8.3 (p-value < 0.05; two-sided hypergeometric test, Fisher exact with Bonferroni correction). Data were categorized into three groups, where DF2 VS DFSCs comprised Group 1, DF8 VS DFSCs comprised Group 2 and DF18 VS DFSCs comprised Group 3.

### 2.4. Quantitative Real Time-PCR

Total RNA was extracted from cells using 200 μL/mL of Trizol (Rionlon, China), according to the manufacturer’s instructions. Thereafter, cDNA (cDNA) was synthesized using the TransScript® First-Strand cDNA Synthesis SuperMix (TransGen, China). Additionally, quantitative Reverse Transcription-polymerase Chain Reaction (qRT-PCR) was conducted using 1 ml of cDNA and the SYBR Green Supermix (TransGen, China), on the FAST 7500 Real-Time PCR System (ABI, USA). All procedures were performed according to the manufacturer’s protocol.

### 2.5. Western blot analysis

Western blot was performed as described previously [3]. Total proteins were extracted using a Total Protein Extraction Kit (Solarbio, China). After standard SDS–polyacrylamide gel electrophoresis and Western blotting, proteins were then visualized using a highly sensitive ECL luminescent liquid (Beyotime, China) then imaged using ChemiScope series 3300 mini-imaging system (Clinx Science, China). The following antibodies were used in the current study; Anti-Id3 (ab236505, Abcam, Dilution: 1:200); Anti-Smad3 (ab40854, Abcam, Dilution: 1:500); Anti-TGF beta 2 (ab205150, Abcam, Dilution: 1:2000); Anti-TGF beta Receptor I (ab31013, Abcam, Dilution: 1:1000); Anti-Bmp4 (ab39973, Abcam, Dilution: 1:500); Anti-MADH7/Smad7 (ab216428, Abcam, Dilution: 1:200); Bmp2 (sc-6895, Santa- Cruz, Dilution: 1:200); Anti-Bambi/NMA (ab203070, Abcam, Dilution: 1:100) and Anti-GAPDH (ab181602, Abcam, Abcam, Dilution: 1:1000). Finally, the protein bands were scanned and analyzed in ImageJ software for gray value.

### 2.6. Osteogenic differentiation

Cells were induced with osteogenic inducing medium containing 10% FBS, 10 mM b-glycerophosphate (Sigma), 100 nM dexamethasone (Sigma), 50 mg/ml ascorbic acid and 0.01 mM 1,25-dihydroxy-vitamin D3 (Sigma) [18] for 14 d. Medium was changed every 2 d. After 14 d of culture, induced DFCs were washed 3 times in PBS after being fixed in 4% paraformaldehyde for 10 minutes and then incubated in 0.1% alizarin red solution (Sigma) in Tris-HCl (pH 8.3) at 37°Cfor 30 minutes. Cells were washed and observed using a phase-contrast inverted microscope (Nikon, Japan).

### 2.7. In vivo *implantation*

#### 2.7.1. Scaffold preparation

Treated Dental Matrix (TDM) was prepared following a previously published protocol [18]. The mandible molar of 3-month-old Sprague–Dawley (SD) rats was extracted. The tooth crown was then cut off and the root was retained. Soft tissues connected to the root were cleaned. The cementum, dental pulp and part of the dentin were also removed. The remaining dentin matrix was immersed in deionized water and thoroughly cleaned through ultrasonic vibration for 5 hours (5 minutes/hour). And then using 17% and 5%EDTA treated TDM separately and cleaned the TDM with deionized water 5 minutes after every step. Then TDM was immersed in PBS supplemented with 100 units/ml penicillin-streptomycin for 72 hours and cleaned with deionized water for 5 minutes. Finally, TDM was stored in α-MEM medium supplemented with 100 units/ml penicillin-streptomycin at 4°C standby applications. The prepared TDM was then placed into a 6-well plate, and combined with cells. The scaffold was considered ready when the TDM was completely covered with cells.

#### 2.7.2. Socket preparation

After anesthesia, 3-month-old SD rats were subjected to anesthesia and the first molar was extracted. The socket was then cleaned, after which the scaffold was implanted. Further, the socket was closed using gingiva. Rats were then sacrificed after 4 weeks, and the implanted parts were extracted for further analysis.

### 2.8. Hematoxylin and eosin staining

Previous implanted parts were fixed with 4% paraformaldehyde overnight at 4°C, demineralized with 10% EDTA (pH 8.0), and embedded in paraffin. Then HE staining was conducted flowing to standard protocols [19]. Paraffin section was treated with dimethyl benzene, ethyl alcohol, 95%, 85% and 75% alcohol. The sections were stained with hematoxylin solution for 5 min. Sections were rinsed with distilled water, and then placed in 1% acid ethanol (1% HCl in 70% ethanol), followed by rinsing in distilled water. Sections were then stained with eosin solution for 30 sec then rinsed using distilled water. Further, sections were treated with 85% alcohol, 95% alcohol, ethyl alcohol, and dimethylbenzene. Stained sections were then examined and photographed using an OLYMPUS IX71 microscope (Tokyo, Japan).

### 2.9. Statistical analysis

Quantitative data were presented as mean SD. Statistical analysis was conducted using Graph-Pad Prism software Version 9.0 (Graph-Pad software, Inc., La Jolla, CA, USA). A Student-Newman-Keuls test was performed to determine differences between groups. A value of P < 0.05 was considered statistically significant.

## 3. Results

In this part, three sub-clones were selected from DFSCs. The differentiation capability of sub-clones was compared to DFSCs *in vitro* and *in vivo*. We also used gene expression array, GO enrichment and KEGG enrichment to explore the effect of heterogeneity on the differentiation potential of sub-clones.

### 3.1. Selection of sub-clones

At the beginning, there were 18 subclones were selected from rat-DFSCs. However, most of the subclones cannot be passed over 5 passages. Finally, three sub-clones (DF2, DF8, and DF18) (Figure 1(b)) were selected from heterogeneous DFSCs according to their different morphology, proliferation potential (Figure 1(c)) and hierarchical clustering (Figure 1(d)).

### 3.2. Sub-clones showed different gene expression patterns, compared with the DFSCs

**Figure 2.**
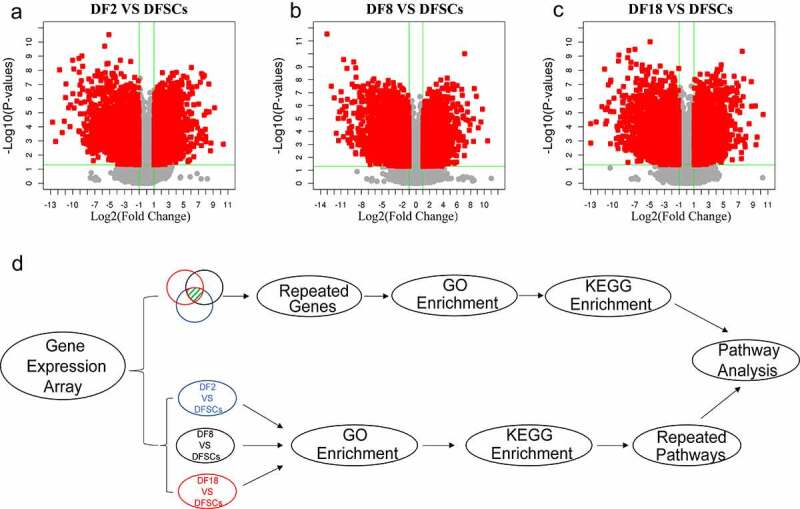
The Volcano plot of differentially expression genes between (a) DF2 and DFSCs, (b) DF8 and DFSCs. (c) Experimental design and flow diagram. The upper part showed the DEGs were selected from three groups and then performed enrichment analysis. The lower part showed the enrichment analysis was firstly performed in different groups separately and then analyzed pathways

Volcano plots showed that there are more down-regulated genes in the sub-clones, compared with the heterogeneous DFSCs (Figure 2). A total of 4212 DEGs in Group 1 (DF2 VS DFSCs), including 2057 upregulated and 2155 down-regulated genes. In addition, Group 2 (DF8 VS DFSCs) showed a total of 4618 DEGs, including 2198 upregulated and 2420 down-regulated genes. Moreover, Group 3 (DF18 VS DFSCs) had a total of 4345 DEGs, including 2110 upregulated and 2235 down-regulated genes (Table 1).

**Table 1.** Number of differential expression genes

Comparison	Differential Genes	Up	Down
DF2 VS DFSCs	4212	2057	2155
DF8 VS DFSCs	4618	2198	2420
DF18 VS DFSCs	4345	2110	2235

**Figure 3.**
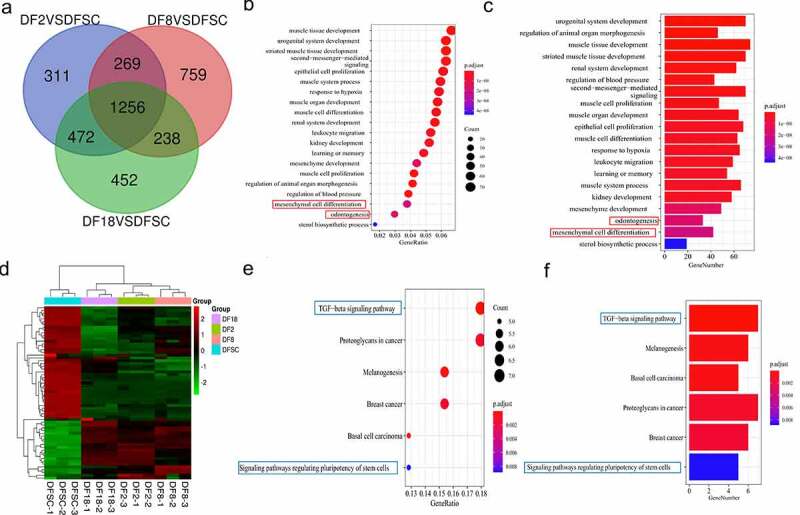
Screening for repeated differentially expressed genes in three groups. (a) Venn diagram. (b) Bubble plot showing results of GO term enrichment analysis of repeated differentially expressed genes. (c) Bar plot showing results of GO term enrichment analysis of repeated differentially expressed genes. (d) Heatmap of the differentially expressed genes in targeted biological processes. (e) Bubble plot showing results of KEGG term enrichment analysis of repeated differentially expressed genes. (f) Bar plot displaying results of KEGG term enrichment analysis of repeated differentially expressed genes. Red frame shows the targeted biological processes in this study. Blue frame shows the targeted signaling pathways in this study

A total of 1256 common DEGs were identified in the three groups (Figure 3(a)). GO enrichment terms of Biological Process (BP) showed that the significant DEGs were highly enriched in development. Notably, the present study mainly focused on two biological processes: *mesenchymal cell differentiation* and *odontogenesis*, based on the objectives of the study and the origin of DFSCs (Figure 3(b,c)). The findings showed that the p-values of both *mesenchymal cell differentiation* and *odontogenesis* were significantly less than 0.01, indicating that there was a significant difference ub the two processes between the sub-clones and DFSCs.

The findings also showed that 64 genes were involved in *mesenchymal cell differentiation* and *odontogenesis*. In addition, a clustering heatmap was used to illustrate the DEGs in the two biological processes between the sub-clones and DFSCs (Figure 3(d)). Notably, the sub-clones exhibited differential gene expression profiles compared with the DFSCs. KEGG pathway enrichment analysis was conducted to explore the possible mechanism underlying changes in gene expression in the sub-clones. Findings from KEGG pathway analysis showed that the TGF-β signaling pathway and signaling pathways regulating the pluripotency of stem cells were implicated in regulation of *mesenchymal cell differentiation* and *odontogenesis* (Figure 3(e,f)).

### 3.3. The three groups showed differential gene expression patterns in the two biological processes

Although the two signaling pathways are involved in changes in the properties of sub-clones, GO and KEGG enrichment of previous method (Figure 2(d) upper part) may have omitted some meaningful pathways. This is because each group had several DEGs that were not included in GO enrichment analysis. Therefore, further analysis was conducted to validate the above results (Figure 2(d) lower part).

**Figure 4.**
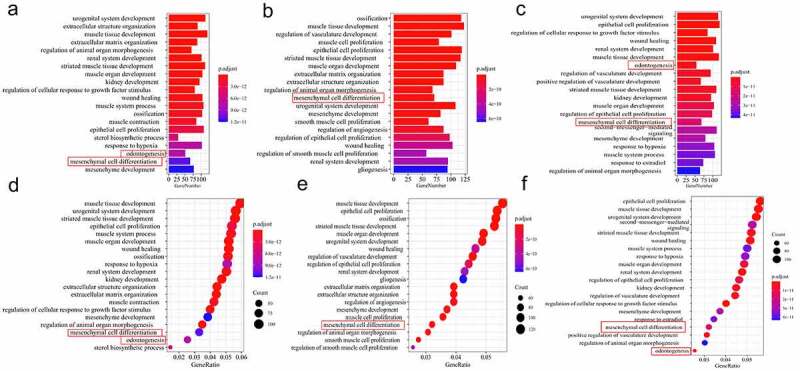
Screening for repeated differentially expressed genes in each group. Bubble plot for GO term enrichment of (a) DF2 VS DFSCs, (b) DF8 VS DFSCs, (c) DF18 VS DFSCs. Bubble plot for GO term enrichment of differential genes of (d) DF2 VS DFSCs, (e) DF8 VS DFSCs, (f) DF18 VS DFSCs. Red frame showed the targeted biological processes in this study

**Figure 5.**
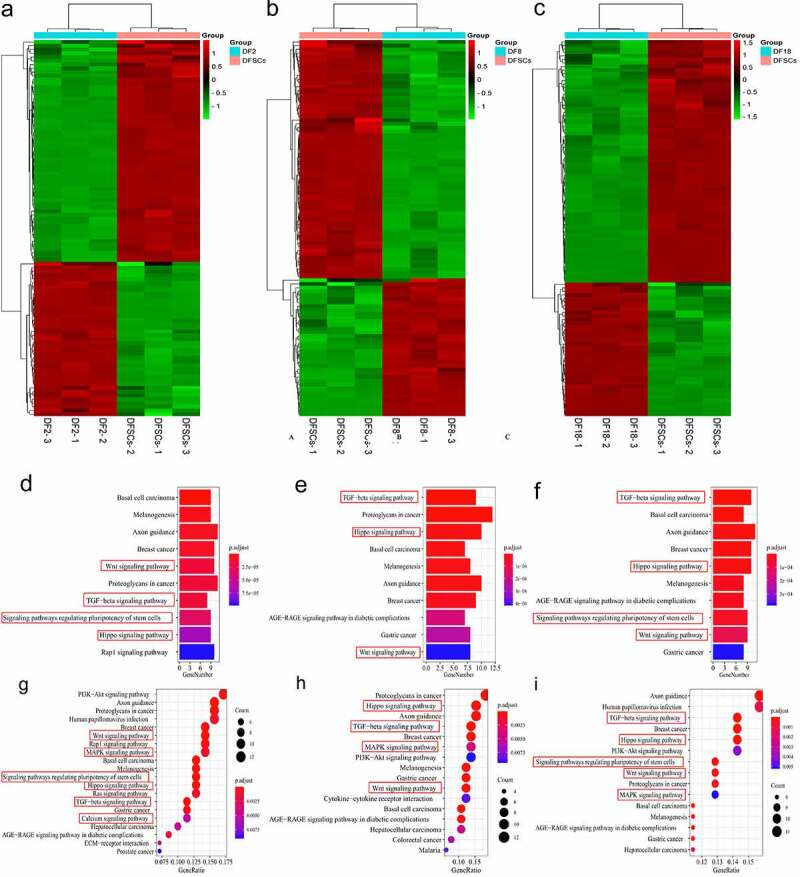
Differential genes expression screening respectively on signaling pathways. Heatmap of the differential genes in targeted biological processes (a) DF2 VS DFSCs; (b) DF8 VS DFSCs, (c) DF18 VS DFSCs. Bubble plot for KEGG term enrichment of (A) DF2 VS DFSCs, (B) DF8 VS DFSCs, (C) DF18 VS DFSCs. Bubble plot for KEGG term enrichment of differential genes of (d) DF2 VS DFSCs, (e) DF8 VS DFSCs, (f) DF18 VS DFSCs. Red frame showed the targeted signaling pathway in this study

Enrichment analysis for the biological process was conducted on the DEGs in the three groups separately. Results from the gene ontology enrichment analysis for the sub-clones and DFSCs are shown in Figure 4. Notably, DEGs were enriched in the top 20 biological processes, including *odontogenesis* and *mesenchymal cell differentiation*, in the three groups. The findings showed that 117 genes were involved in the two processes in Group 1 (51 in *odontogenesis* and 66 in *mesenchymal cell differentiation*). In Group 2, a total of 118 genes were involved in these two processes (47 in *odontogenesis* and 71 in *mesenchymal cell differentiation*); however, *odontogenesis* was not enriched in the top 20 BP terms (Figure 4(b,e)). A total 122 genes were involved in these two processes (54 in *odontogenesis* and 68 in *mesenchymal cell differentiation*) in Group 3 (Figure 5(c,f)).

A heatmap of DEGs in three groups showed differential gene expression patterns between the sub-clones and heterogenous DFSCs. However, the gene expression patterns in different sub-clones were not consistent because more DEGs were included (Figure 5(a–c)).

### 3.4. Several signaling pathways were involved in changes in differentiation of sub-clones

DEGs in the two biological processes were separately analyzed through pathway enrichment analysis using the KEGG database. The findings showed that a total of 15 pathways were enriched in Group 1. Notably, 6 out of 15 pathways were related to cell differentiation, including TGF-β signaling pathway, WNT signaling pathway, signaling pathways regulating the pluripotency of stem cells, Hippo signaling pathway, calcium signaling pathway, and MAPK signaling pathway (Figure 5(d,g)). In Group 2, a total of 15 pathways were enriched, and 4 pathways out of the 15 were related to cell differentiation and were significantly enriched, including the TGF-ß signaling pathway, WNT signaling pathway, Hippo signaling pathway and MAPK signaling pathway (Figure 5(e,h)). Moreover, a total of 15 pathways were enriched in Group 3, 5 pathways out of the 15 were implicated in cell differentiation and were also significantly enriched. These pathways included TGF-ß signaling pathway, WNT signaling pathway, signaling pathways regulating the pluripotency of stem cells, MAPK signaling pathway and Hippo signaling pathway (Figure 5(c,i)).

### 3.5. Cross-talk in pathways regulated changes in differentiation

**Figure 6.**
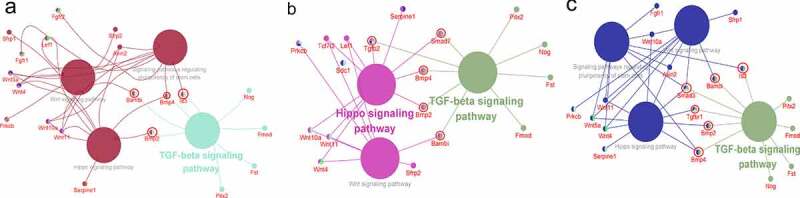
Interaction network analysis to DRGs by Cytoscape (a) DF2 VS DFSCs group, TGF-β signaling pathway worked with WNT signaling pathway, signaling pathways Regulating the Pluripotency of Stem Cells and Hippo pathway through Id3, Bmp4, Bmp2 and Bambi. (b) DF8 VS DFSCs group, TGF-β signaling pathway worked with WNT signaling pathway and Hippo pathway through Smad7, Bmp4, Bmp2, TGFβ2 and Bambi. (c) DF18 VS DFSCs group, TGF-β signaling pathway worked with WNT signaling pathway, signaling pathways Regulating the Pluripotency of Stem Cells and Hippo pathway through Id3, Bmp4, Bmp2 and Bambi, Smad3 and TGFβR1. (The green color represented the most enriched pathways)

Interactions of significant DEGs were explored through a network diagram, constructed using Cytoscape (Figure 6). Network analysis showed that TGF-β was the most dominant signaling pathway. However, the WNT signaling pathway, Hippo signaling pathway and signaling pathways regulating the pluripotency of stem cells also played an important role in the decrease of differentiation. In Group 1, the TGF-β signaling pathway functioned synergistically with Hippo signaling pathway through Bmp2 and Bmp4 to regulate differentiation. Additionally, the TGF-β signaling pathway functioned along with the WNT signaling pathway through Bambi. The findings showed that the TGF-β signaling pathway played a synergistic role with signaling pathways regulating the pluripotency of stem cells, through Bmp4. In Group 2, the TGF-β signaling pathway played a synergistic role with the Hippo signaling pathway through Bmp2, Smad7 and Bmp4 to modulate differentiation. Additionally, TGF-β signaling pathway worked together with the WNT signaling pathway through Bambi. In Group 3, the TGF-β signaling pathway functioned synergistically with the Hippo signaling pathway through Bmp2, Smad3 and Bmp4 to modulate differentiation. Furthermore, TGF-β signaling pathway regulated differentiation by working synergistically with the WNT signaling pathway through Bambi and Smad3. TGF-β signaling pathway also worked synergistically with Signaling pathways regulating the pluripotency of stem cells through Bmp4 and Smad3 to modulate differentiation in Group 3.

### 3.6. Validation of gene and protein expression

**Figure 7.**
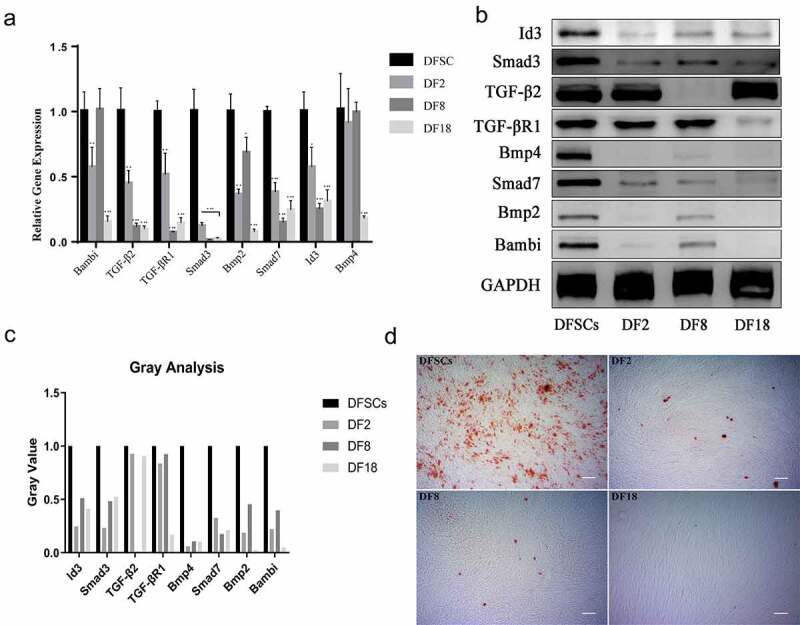
Validation of microarray results by qRT-PCR (a) and Western blot (b). Statistical significance used in this figure: *P < 0.05, **P < 0.01 and ***P < 0.001; ns represented no statistically significant. Error bar indicated SD (n = 3). (c) Gray analysis for Western blot. The Gray value of subclones was significantly lower than DFSCs. (d) Osteogenesis Differentiation. Calcium nodules were visualized using alizarin red after osteogenesis induction of DFSCs and sub-clones. There were fewer calcium nodules in the sub-clones. (Scale bar: 100 mm)

Enrichment results were validated through qRT-PCR (Figure 7(a)). The findings showed significant decrease in expression of Bmp2, Bmp4, Smad7, TGF-β2, TGF-βR1, Smad3, and Bambi in the three sub-clones, compared with the expression level in the DFSCs. However, there was no significant difference in expression of Bambi in the DF8 VS DFSCs category. Moreover, differential expression of Bmp4 was only significant in the DF18 VS DFSCs group.

Western blot analysis was used to validate genes expression in the enriched pathways (Figure 7(b,c)). The results showed a decrease in expression levels of proteins in the three sub-clones, compared with the protein levels in the DFSCs. However, expression level of TGF-β2 and TGF-βR1 proteins was not consistent with their gene expression results. Notably, TGF-β2 was only down-regulated in DF8 whereas TGF-βR1 was only down-regulated in DF18.

### 3.7. Subclones showed reduced in vivo differentiation and odontogenesis capacity compared with DFSCs

**Figure 8.**
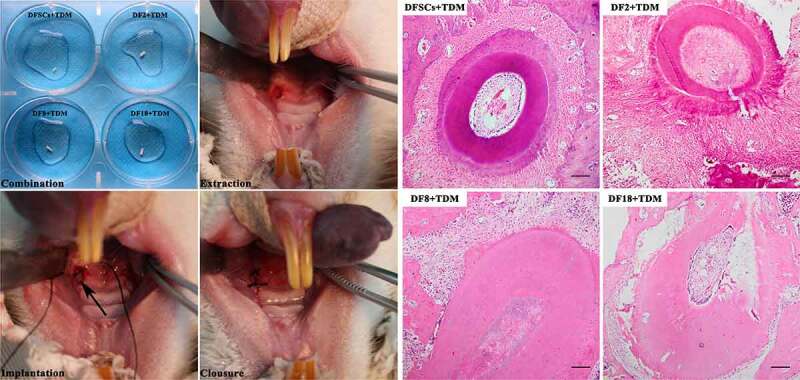
*In vivo* implantation. Left photos: DFSCs and three sub-clones were combined with TDM, and the scaffolds were implanted into sockets. Right photos: HE staining for different scaffolds. The new bone formation was observed in DFSCs + TDM group and DF2+ TDM group. (Scale bar: 100 μm)

To explore the differentiation capacity of the three sub-clones *in vitro*. The osteogenesis differentiation was conducted to evaluate the differentiation potential of sub-clones. The results show that fewer mineralized nodules can be found in sub-clones (Figure 7(d)). In order to explore the differentiation capacity of the three sub-clones *in vivo*, TDM combined with sub-clones were implanted into the socket of the SD rats (Figure 8). *In vivo* results showed that the DFSCs +TDM regenerated periodontal-like tissues including bone and fiber tissues. However, the three sub-clones did not regenerate into periodontal-like tissues and the findings showed that only DF2 + TDM formed partial bone-like and fiber-like tissues.

## 4. Discussion

Multipotential differentiation is one of the main characteristics of stem cells. Therefore, any changes in multipotency of stem cells can cause unpredictable outcomes. Notably, external environment, including induction of differentiation, stress [20] and radiation [21], can affect differentiation ability of stem cells. Moreover, internal environment can affect the differentiation ability of stem cells. Heterogeneity is a special feature of stem cells and it affects homeostasis in their internal environment [22]. In the current study, homeostasis of DFSCs was artificially destroyed. The findings showed that the three sub-clones underwent continuous proliferation. In addition, several biological processes, including differentiation potential, were dysregulated in the three sub-clones. *In-situ* implantation analysis showed that the sub-clones lost their regeneration capacity, owing to differentiation capability changes. Although artificial damage of the heterogeneous microenvironment was an extreme measure and loss of heterogeneity in practice is a very slow process, the findings showed dysregulation of internal homeostasis in stem cells. Therefore, our results indicated that further studies should be conducted on stem cells, selected by surface markers. Moreover, the selection method interfered with homeostasis, which may have led to changes in the properties of stem cells.

In the current study, TGF-β signaling pathway and signaling pathways regulating the pluripotency of stem cells were initially shown to be responsible for changes in differentiation capacity. However, TGF-β was the most dominant signaling pathway. TGF-β signaling pathway is involved in several biological processes, including cell proliferation, migration, and differentiation [23,24]. This indicates that TGF-β signaling pathway is implicated in modulating heterogeneity of stem cells [25]. Moreover, analysis of genes and individual analysis of sub-clones showed that the TGF-β signaling pathway played an important role in changes in cell differentiation. In addition, a decrease in activity of the TGF-β signaling pathway led to decrease in differentiation capacity of sub-clones. In addition, signaling pathways regulating the pluripotency of stem cells were significantly enriched in the two methods. DFSCs exhibited certain properties of embryonic stem cells. Therefore, down-regulation of signaling pathways regulating the pluripotency of stem Cells indicated that the sub-clones had lost their original embryonic properties. This reduction further abrogated the differentiation of sub-clones.

Further analysis (Figure 2(d) lower part) similarly showed that TGF-β was the most dominant signaling pathway. However, the findings showed that Hippo and WNT signaling pathways were also involved in loss of differentiation capacity of the sub-clones. Hippo signaling pathway is a conserved network that plays a vital role in maintaining stemness and proliferative ability of stem cells [26,27]. It thus played an important role in modulating homeostasis in organs [28]. A unique feature of the Hippo signaling pathway is that morphological changes in cells can alter the activity of the signaling pathway [29]. In the current study, three clones were selected from heterogeneous DFSCs with different morphologies, indicating changes in cytoskeleton. Although the expression levels of its key regulator, YAP/TAZ was normal, the Hippo signaling pathway was still affected, further decreasing ability of differentiation. WNT signaling pathway is also a conserved network that plays a contradicting role in regulating stem cells. WNT signaling pathway maintains embryonic and stemness characteristics of stem cells, thus maintaining their pluripotency [30]. On the contrary, WNT signaling pathway promotes differentiation ability of stem cells in different induction environments [31]. In the current study, the WNT signaling pathway was not inhibited by increased DKK1 and differentiation ability of the three clones was decreased. These findings show that showed damage of the heterogeneous environment deactivated the signaling pathways in the three sub-clones, resulting in a decrease in differentiation capacity.

Moreover, the findings of the present study showed that four vital signaling pathways are involved in loss of differentiation capacity in the three sub-clones. Further analysis indicated that cross-talk in signaling pathways was the key regulator rather than activity of an individual signaling pathways. In addition, several DEGs were enriched in the TGF-β signaling pathway, compared with the other three pathways. The findings of the study also showed cross-talk between TGF-β signaling pathway and the other three pathways. Notably, network analysis and validation analysis showed cross-talk between TGF-β signaling pathway and signaling pathways regulating the pluripotency of stem cells, Hippo signaling pathway and WNT signaling pathway, through Bmp2, Bmp4 and Bambi. The three genes were significantly down-regulated in the three clones. In addition, the findings showed that these linker genes were involved in maintaining stemness and regulating differentiation of cells by affecting the corresponding signaling pathways [32]. For instance, analysis showed that Bmp2 and Bmp4 were important regulators of cross-talk between TGF-β and Hippo signaling pathways, at multiple levels [33]. In the current study, low expression of Bmp2 and Bmp4 that linked the TGF-β and Hippo signaling pathways was observed, resulting in deactivation of both pathways. Bambi is a BMP and Activin Member Bound Inhibitor implicated in terminal differentiation by regulating activity of TGF-β signaling pathway [34]. Bambi works synergistically with Bmp2 to induce activation of WNT signaling pathway [35]. This study showed low expression of Bambi that linked TGF-β and Hippo signaling pathways, resulting in inhibition of both pathways. Notably, apart from the four targeted pathways, changes in the other signaling pathways were not consistent in the three clones. In addition, expression of the linker genes varied in three clones. Therefore, further studies should explore more common factors in the three sub-clones.

## 5. Conclusion

**Figure 9.**
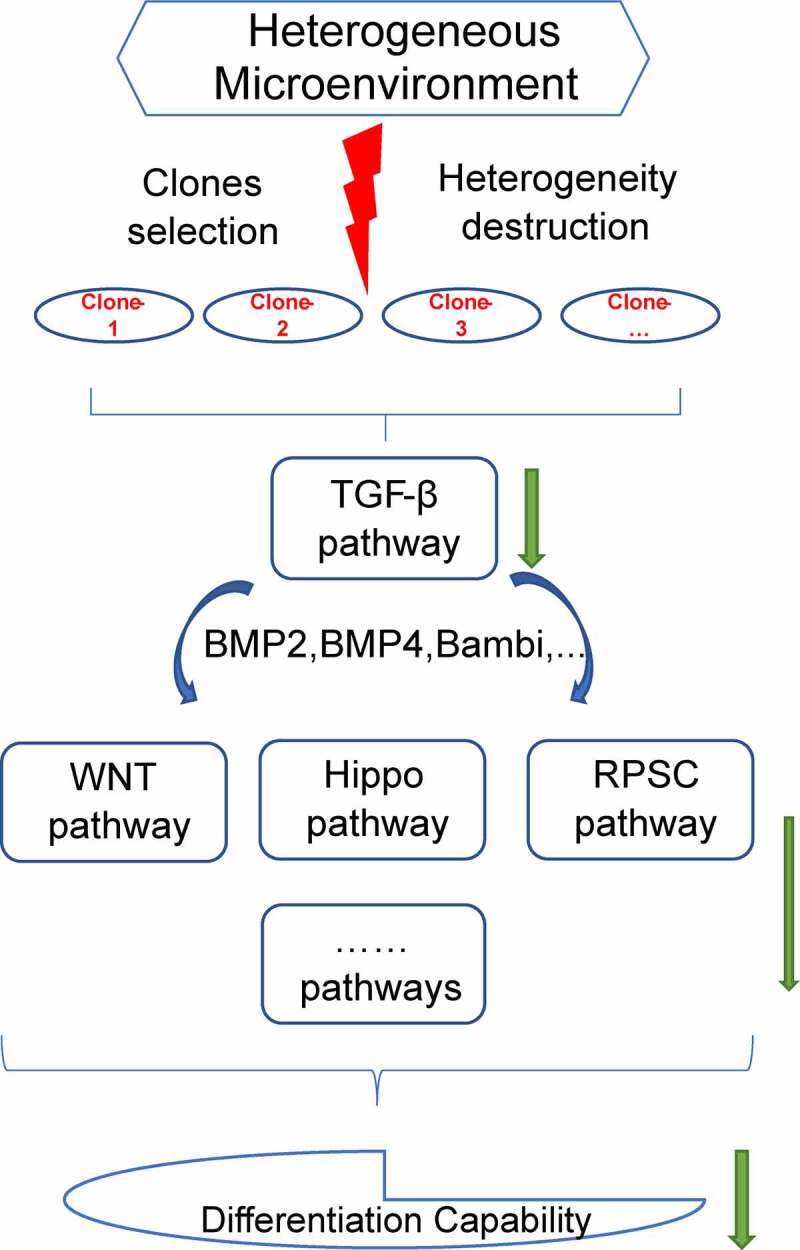
Study outcome. Selection of sub-clones destructed the heterogeneous microenvironment of DFSCs. The destruction of heterogeneity down-regulated TGF-β signaling pathway. TGF-β signaling pathway cross talked with Hippo signaling pathway, WNT signaling pathway, signaling pathways regulating the pluripotency of stem cells and other pathways inhibited differentiation capability of sub-clones

In summary, the findings of the current study showed that loss of heterogeneity can affect the differentiation capacity of the sub-clones, although sub-clones exhibited a self-renewal capability. In addition, TGF-β signaling pathway modulated this decrease in differentiation potential through cross-talk with Hippo signaling pathway, WNT signaling pathway, signaling pathways regulating the pluripotency of stem cells and other pathways (Figure 9). These findings indicated the effects of heterogeneity on differentiation, providing a basis for application of stem cells.
